# The co-occurrence of Kikuchi–Fujimoto disease and systemic lupus erythematosus: a case report

**DOI:** 10.1186/s13256-023-04186-4

**Published:** 2023-10-27

**Authors:** Maysam Yousefi, Mohammad Rezaei Zadeh Rukerd, Hanieh Binafar, Sahar Shoaie, Hanieh Mirkamali, Pouria Pourzand, Roxana Kaveh

**Affiliations:** 1 Infectious Diseases Research Center of Tropical and Infectious Diseases, Kerman University of Medical Sciences, Kerman, Iran; 2 Gastroenterology and Hepatology Research Center, Institute of Basic and Clinical Physiology Sciences, Kerman University of Medical Sciences, Kerman, Iran; 3 Student Research Committee, School of Medicine, Kerman University of Medical Sciences, Kerman, Iran; 4 Department of Internal Medicine, Afzalipour Hospital, Kerman University of Medical Sciences, Kerman, Iran; 5 Department of Emergency Medicine, School of Medicine, University of Medicine, Minneapolis, USA

**Keywords:** Kikuchi–Fujimoto disease, Systemic lupus erythematosus, Iran

## Abstract

**Background:**

Kikuchi–Fujimoto disease is an uncommon systemic disease that mostly affects young women. Kikuchi–Fujimoto disease typically manifests as necrotizing lymphadenopathy, which frequently follows by a fever; however, Kikuchi–Fujimoto disease occurs rarely in extranodal regions. One of the most important accompaniments of Kikuchi–Fujimoto disease is its connection with autoimmune diseases such as systemic lupus erythematosus. This case presents a simultaneous occurrence of Kikuchi–Fujimoto disease with liver involvement and systemic lupus erythematosus in a young female patient.

**Case presentation:**

We present a rare case of a 20-year-old white woman who presented with fever, joint pains, myalgia, and shortness of breath. Initial hospitalization and treatment for fever of unknown origin did not yield improvement. Physical examination revealed cervical and supraclavicular lymphadenopathy, and laboratory investigations showed abnormal blood counts, elevated inflammatory markers, and positive autoimmune serologies. Imaging studies revealed bilateral pleural effusion and liver lesions. Lymph node biopsy confirmed the diagnosis of Kikuchi–Fujimoto disease, and liver biopsy showed extranodal involvement. The patient was diagnosed with Kikuchi–Fujimoto disease-associated systemic lupus erythematosus and treated with hydroxychloroquine and corticosteroids. The patient showed gradual resolution of symptoms and lymphadenopathy with treatment.

**Conclusion:**

Kikuchi–Fujimoto disease is a rare systemic condition primarily impacting young females. It is characterized by necrotizing lymphadenopathy, often accompanied by fever. Although Kikuchi–Fujimoto disease is predominantly seen in the lymph nodes, occurrences in non-nodal areas are infrequent. When diagnosing Kikuchi–Fujimoto disease, it is essential to screen patients for systemic lupus erythematosus. In this particular case, we observed liver involvement along with the presence of both Kikuchi–Fujimoto disease and systemic lupus erythematosus.

## Background

Kikuchi–Fujimoto disease (KFD), also known as histiocytic necrotizing lymphadenitis, is a rare systemic disease that typically affects among young Asian women; however, it has been widely reported around the world [1, 2]. KFD can affect anyone at any age, but it typically affects people under the age of 40 years [1, 3]. Although Kikuchi and Fujimoto first described KFD in 1972, its etiology is still not completely understood [1]. However, there are two major hypotheses regarding the pathophysiology of the KFD—autoimmune disorders and infectious agents [4].

KFD typically presents as tender, swollen, and subacute necrotizing lymphadenopathy, which are frequently accompanied by a low-grade fever and fatigue [1, 5]. Headache, nausea, vomiting, malaise, weight loss, arthralgia, myalgia, night sweats, rash, and thoracic/abdominal pain are additional symptoms [6, 7]. KFD occurs rarely in extranodal regions, such as the skin, bone marrow, and liver [1, 6]. Histological evaluation of lymph nodes typically revealed a partially preserved structure with follicular hyperplasia, in addition to well-defined zones of necrosis [1]. The majority of patients have normal laboratory results [1]. Anemia, elevated erythrocyte sedimentation rate (ESR), C-reactive protein (CRP), serum lactate dehydrogenase (LDH), aminotransferases, and leukopenia are common findings in others [1, 3].

Due to the disease’s nonspecific symptoms, it can easily be misdiagnosed with other infectious, autoimmune, or malignant diseases [6, 7]. Although KFD is a self-limited disease, a prompt diagnosis is necessary to avoid aggressive treatment [1]. In this study, we present a unique and rare case of KFD with liver involvement, which occurred concurrently with systemic lupus erythematosus (SLE). Our report not only emphasizes the diagnostic and therapeutic strategies employed but also contributes to the existing knowledge by providing valuable insights into this uncommon co-occurrence. We believe that this case study will enhance our understanding of the disease and contribute to the existing body of knowledge in this field.

## Case presentation

A 20-year-old white woman presented with 4-week history of fever, chills, generalized joint pains, photosensitivity, fatigue, and myalgia. Since a month previously, the patient had complained of morning stiffness lasting more than an hour, as well as progressive shortness of breath in the last 3 days. She reported a recent history of recurrent and fleeting painless oral ulcers over the past month. No gastrointestinal manifestations were observed in the patient, such as abdominal pain, rectal bleeding, sensations of nausea and vomiting, and changes in bowel habits. There was no history of weight loss, night sweats, or specific skin rashes. Additionally, she denied any symptoms of Raynaud’s phenomenon affecting her extremities. There were no recent reports of neuropsychiatric symptoms exhibited by the patients. She claimed not to be using tobacco, alcohol, or illegal drugs, as well as having a history of any diseases. She was a housewife with no prior contact to anyone exhibiting comparable symptoms. Her family history and recent travel within the past 6 months were not contributing factors. According to national protocol, the patient was current on all required vaccinations.

The patient had spent 3 days in the hospital 2 weeks prior to this administration, at which time she was diagnosed with fever of unknown origin (FUO) and was treated with metronidazole and cefixime. However, despite her management, symptoms did not improve and gradually worsened over the past weeks before current admission.

At the admission, physical examination showed body temperature of 39 °C (axillary), heart rate of 75 beats/min, respiratory rate of 15 breaths/min, blood pressure of 110/75 mmHg, and oxygen saturation of 97% while breathing ambient air. She appeared acutely ill and weak, but she was alert and oriented, and she responded appropriately to questions. Physical examination also revealed diffused rubbery, soft, and mobile right cervical and supraclavicular lymphadenopathy with a diameter of 2 cm, with no inflammatory signs. On palpation of the abdomen, there was no sign of hepatomegaly or splenomegaly. On oral physical examination, a solitary 2 cm lesion covered by exudates, surrounded by a reddish aura, was observed in the buccal mucosa, without any signs of blistering. During the skin examination, no discernible indications of typical skin lesions, such as a malar rash, were observed. There was no evidence of current acute inflammation in joints. No signs of genital aphthous ulcer were detected.

The results of a complete blood count (CBC) revealed a white blood cell count of 3.1 × 10^9^/L, a hemoglobin level of 9 g/dL, and a platelet count of 320 × 10^9^/L. The serum level of aspartate aminotransferase (AST) was 22 IU/L (normal < 40 IU/L), alanine aminotransferase (ALT) 29 IU/L (normal < 40 IU/L), and alkaline phosphatase (ALP) 116 IU/L (normal < 206 IU/L). The results of the laboratory analysis showed that the patient’s ESR was 74 mm/hour, CRP was 69 mg/L, and LDH was 1324 IU/L (normal values are between 140 and 280 IU/L). On subsequent analysis, no microorganisms were grown in the patient’s blood and urine cultures.

The HITACHI Roche Cobas C311 chemistry analyzer was used to detect the serologies with an enzyme-linked immunosorbent assay (ELISA). Her Coombs Wright, 2-mercaptoethanol (2ME), perinuclear anti-neutrophil cytoplasmic antibody (p-ANCA), and cytoplasmic anti-neutrophil cytoplasmic antibody (c-ANCA) tests were negative by ELISA. Negative results were found in tests for human immunodeficiency virus (HIV), hepatitis viruses, Epstein–Barr virus (EBV), and cytomegalovirus (CMV). The blood samples specifically analyzed through the venereal disease research laboratory (VDRL) test and fluorescent treponemal antibody absorption (FTA-ABS) test showed negative results. The Tzanck smear performed on the oral ulcer to detect multinucleated giant cells, indicative of herpes simplex virus (HSV), yielded a negative result. She had a positive antinuclear antibody (ANA) titer of 1/1600 with speckled pattern, as well as a positive double-strand DNA antibody (dsDNA) titer of 2211 IU/mL. Upon further examination, the levels of C3 and C4 complements were found to be 73 mg/dL (within the range of 80–160) and 10 mg/dL (within the range of 13–39), respectively. SLE was identified as the underlying medical condition based on criteria established by the Systemic Lupus International Collaborating Clinics [8].

**Fig. 1:**
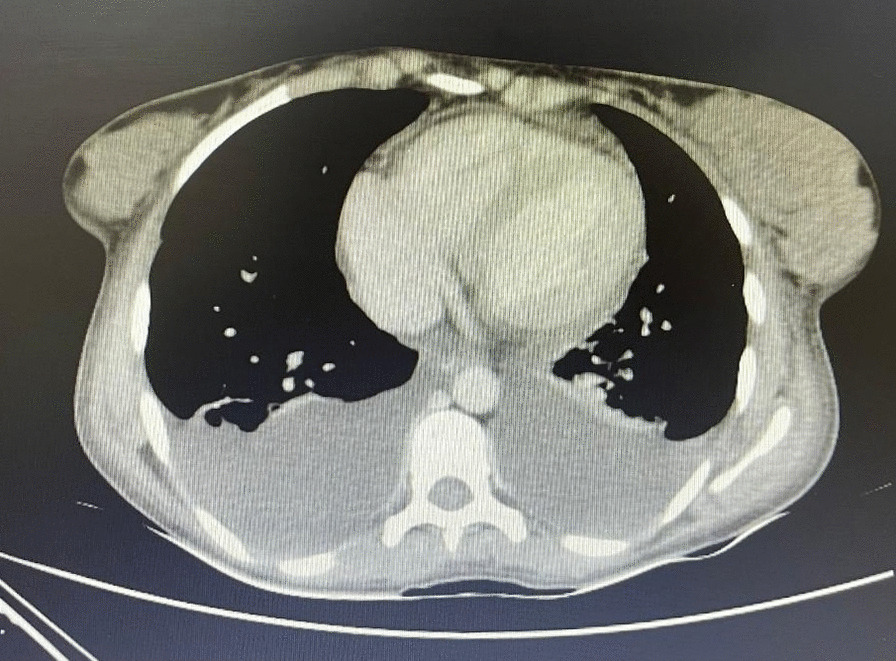
Chest computed tomography scan showing bilateral pleural effusion accompanied by mild pericardial effusion

**Fig. 2:**
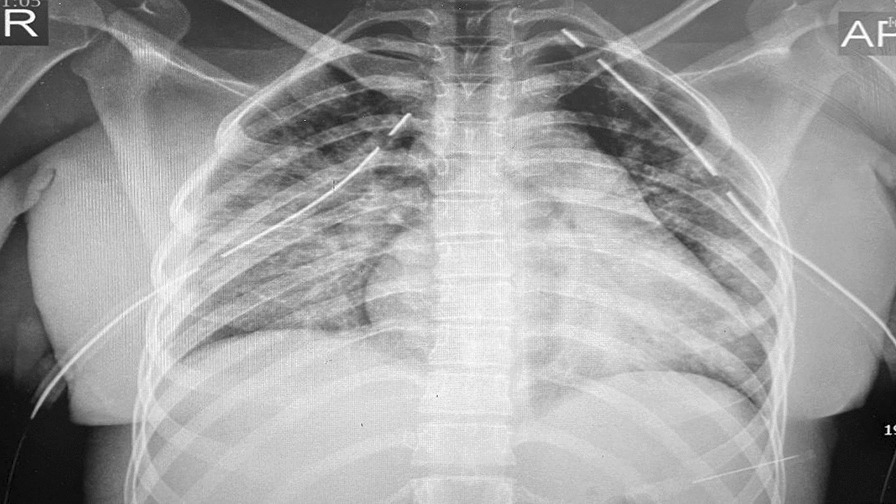
Anterior–posterior view chest x-ray following bilateral chest tube placement

The chest x-ray (CXR) and chest computed tomography (CT) scan showed bilateral pleural effusion (Fig. 1), prompting the placement of bilateral chest tube (Fig. 2). Further examination revealed that the pleural fluid was transudate. An echocardiogram was performed on the patient, and there was evidence of mild pericardial effusion without any endocardial vegetation.

To determine the extent of the lymphadenopathy, additional investigations have been ordered. A neck ultrasonography revealed bilaterally enlarged submandibular and supraclavicular lymph nodes, the largest of which was 2.5 cm in diameter. Although there was no evidence of lymphadenopathy in the abdominopelvic CT scan, a few hypodense lesions in the seventh and sixth segments of the liver were found.

**Fig. 3:**
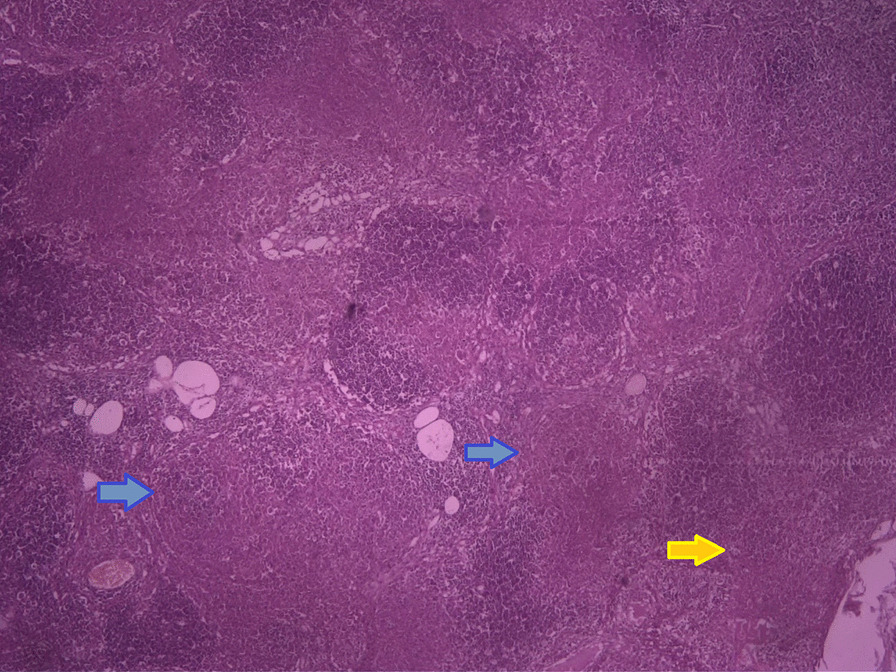
Microscopic examination (×40 magnification) of the lymph node specimen displaying preserved structure with follicular hyperplasia (blue arrows), infiltration of phagocytic cells, and well-defined coagulative necrosis in the paracortical region (yellow arrow)

It was decided that a lymph node biopsy from several cervical lymph nodes would be performed under ultrasound guidance. Lymph node histopathologic evaluation revealed preserved structure with follicular hyperplasia (blue arrows), as well as necrotizing lymphadenitis with paracortical coagulative necrosis (Fig. 3, yellow arrow). Immunohistochemistry (IHC) analysis of the samples showed that CD68 and CD4 were both expressed. These findings were consistent with KFD. Furthermore, a liver core biopsy from a hypodense lesion revealed focal lobular hepatocyte necrosis and inflammation with histiocytic and neutrophils.

Finally, the patient was diagnosed with KFD-associated SLE with extranodal involvement (liver) and was given hydroxychloroquine (HCQ) and oral corticosteroid (prednisolone) therapy. After 5 days of hospitalization, the patient’s condition was well stabilized with no adverse effects, and she was discharged to outpatient care in good health. After 2 months of treatment, the lymphadenopathies showed a gradual reduction until complete resolution. The patient was observed every 2 months for an average of 6 months, during which time she displayed no symptoms.

## Discussion

The majority of patients with KFD are from Asia, while it is uncommon in the USA [9]. Females are the most commonly affected, with an average age of 21 years and a 2:1 female predominance, as described in our case study [10]. Although KFD is a rare cause of lymphadenopathy, it is a form of benign necrotizing lymphadenitis, which is easily confused with other causes of lymphadenopathy such as tuberculosis and lymphoma. Therefore, the exact incidence of KFD is unknown [9, 11]. According to one study, 40% of cases of KFD were misdiagnosed as lymphoma [7]. Also, our case was a woman who had been hospitalized previously and had been misdiagnosed and treated inappropriately. It is necessary to represent the variable clinical manifestations of KFD to decrease misdiagnoses and unnecessary treatments.

Despite the fact that the exact reason is still a mystery, a number of studies have pointed the finger at viruses such as EBV as a possible contributor [1, 11, 12]. There are also studies and case reports that show a strong link between KFD and autoimmune diseases like SLE, with SLE diagnosed before, after, or even concurrently with KFD [13, 14]. At the time of KFD diagnosis, it is important to assess patients for SLE and ensure ongoing monitoring by a rheumatologist to exclude the emergence and advancement of SLE, an important condition associated with significant physical impairments among women [7, 15, 16]. In our research, both KFD and SLE were identified at the same time during the hospitalization process.

Clinically, fever is usually the first symptom in KFD and can be accompanied by upper respiratory symptoms (30–50%), weight loss, night sweats, nausea, vomiting, sore throat, arthralgia, splenomegaly, and rash [1, 17]. The most common clinical feature is unilateral tender lymphadenopathy involving the jugular and posterior cervical lymph nodes [18]. Rarely does it manifest in organs other than lymph nodes, the most common of which is the skin, with the bone marrow and liver also being possible sites [1, 5, 6, 19, 20]. Our patient had enlarged lymph nodes in both the submandibular and supraclavicular regions, fever, chills, arthralgia, photosensitivity, myalgia, morning stiffness, dyspnea, pleural effusion, as well as hypodense lesions in liver, as an extranodal involvement of KFD.

Laboratory findings in KFD are nonspecific, including findings of anemia, leukopenia, thrombocytopenia, and elevations in liver enzymes, LDH levels, and ESR rate [21]. Typically, imaging reveals enlarged enhancing lymph nodes with necrosis; however, it can provide useful evidence but not a definitive diagnosis [10]. Following the exclusion of other diseases, KFD is diagnosed based on histopathologic findings such as follicular hyperplasia, enlarged paracortical areas, patches of necrosis that are well-defined, and necrotic foci, that obtained from fine-needle aspiration or biopsy of the affected lymph node [1, 21]. Our patient’s laboratory results revealed anemia, leukopenia, elevated LDH and CRP levels, a raised ESR rate, and positive ANA and anti-dsDNA tests. Our patient’s KFD diagnosis was confirmed by both histopathology and IHC.

Lymphadenopathy differential diagnosis includes infectious agents such as tuberculosis, toxoplasmosis, HIV, and EBV; inflammation; SLE; lymphoma; and metastasis. Serologic testing can rule out other causes of lymphadenopathy, such as cat scratch disease or toxoplasmosis, while histological findings can rule out cancer [9].

Generally, KFD is a benign and self-limiting disorder (short inpatient stays with symptomatic treatment). However, for patients with significant systemic symptoms (for example, multiple organ systems failure or immune system dysfunction) and those with associated autoimmune disease such as SLE, appropriate drugs such as non-steroidal anti-inflammatory drugs (NSAIDs), hormones, and immunosuppressant agents should be seriously considered to manage the symptoms [1, 13, 22].

## Conclusion

KFD is an uncommon systemic disease that mostly affects young women. KFD typically manifests as necrotizing lymphadenopathy, which frequently follows by a fever; however, KFD occurs rarely in extranodal regions. At the time of diagnosis, patients with KFD should be tested for SLE. In our case, in addition to KFD liver involvement, the presence of KFD and SLE was also observed.

## Data Availability

Not applicable.

## Abbreviations

KFD: Kikuchi–Fujimoto disease
ESR: Erythrocyte sedimentation rate
CRP: C-reactive protein
LDH: Serum lactate dehydrogenase
SLE: Systemic lupus erythematosus
FUO: Fever of unknown origin
CBC: Complete blood count
AST: Aspartate aminotransferase
ALT: Alanine aminotransferase
ALP: Alkaline phosphatase
ELISA: Enzyme-linked immunosorbent assay
2ME: 2-Mercaptoethanol
HIV: Human immunodeficiency virus
EBV: Epstein–Barr virus
CMV: Cytomegalovirus
HSV: Herpes simplex virus
ANA: Antinuclear antibody
dsDNA: Double-strand DNA antibody
CXR: Chest x-ray
CT: Computed tomography
IHC: Immunohistochemistry
HCQ: Hydroxychloroquine
